# Diffusion tensor imaging of the pelvic floor muscles and pelvic organs: a systematic review

**DOI:** 10.3389/fmedt.2026.1772496

**Published:** 2026-06-19

**Authors:** Yiting Wang, Shaojun Zhang, Yan Chen

**Affiliations:** 1 Shanghai Municipal Hospital of Traditional Chinese Medicine, affiliated to Shanghai University of Traditional Chinese Medicine, Shanghai, China; 2 Jiading Hospital of Traditional Chinese Medicine, affiliated to Shanghai University of Traditional Chinese Medicine, Shanghai, China

**Keywords:** diffusion, diffusion tensor imaging, magnetic resonance imaging, pelvic floor, pelvic floor organ

## Abstract

**Purpose:**

This paper provides a systematic review of the current applications of diffusion tensor imaging (DTI) in the imaging of pelvic floor-related anatomical structures and the assessment of microscopic pathological changes.

**Methods:**

A systematic search of the PubMed, EMBASE, and Cochrane Library databases was conducted, with a focus on studies involving the application of diffusion tensor imaging to pelvic organs and muscles. The search was conducted from the inception of each database through to 11 December 2025. The present study incorporated a range of research methodologies, including randomized controlled trials (RCTs) and observational studies. The screening and reporting phases of the study adhered to the PRISMA guidelines, while the quality assessment was conducted using the Oxford Centre for Evidence-Based Medicine (OEBM) evidence grading tool.

**Results:**

The final sample comprised a total of 30 studies. Of these, two studies reported the application of DTI on the anal canal and its diagnostic value for anal fistula. Five studies demonstrated the ability of DTI to visualize microstructural alterations in endometriosis and uterine fibroids by measuring uterine anisotropy fraction and fibre tracking. A total of 19 studies reported the utilization of DTI applications in the context of prostate and prostate tumor analysis. Four studies have documented its use in healthy pelvic muscles and pelvic floor dysfunction.

**Conclusion:**

The findings suggest that the application of diffusion tensor imaging metrics in the pelvic region may aid in the assessment of tissue microstructural disruption and the distinction between benign and malignant or inflammatory lesions through relevant parameters, thereby enhancing diagnostic specificity. As diffusion tensor imaging becomes increasingly utilized in imaging for treatment guidance, it is imperative to develop a comprehensive understanding of its application in the pelvic region.

## Introduction

High-contrast magnetic resonance imaging (MRI) can capture detailed anatomical images with high resolution and provide valuable microstructural insights. Various MRI techniques are available for examining different parts of the body, depending on their specific characteristics (1). Diffusion MRI, in particular, is used to assess molecular diffusion in biological tissues. This technique evaluates the directionality of water diffusion in tissues to gain a deeper understanding of their microstructure and microdynamics (2). It is currently the only method capable of measuring the dispersion and activity of water molecules *in vivo* (3). Diffusion MRI encompasses diffusion-weighted imaging (DWI) and diffusion tensor imaging (DTI), with DTI being an advancement of DWI. DWI focuses on imaging the diffusion of water molecules, while DTI extends this by incorporating the direction of water molecule movement to produce more detailed images (4).

The fractional anisotropy (FA), apparent diffusion coefficient (ADC) and the mean diffusion coefficient (MD) are the most commonly used metrics in DTI to capture information about the primary direction of localized diffusion. FA measures the variation in the directionality of water molecule movement, with a value of “zero” indicating complete isotropy and “one” indicating strong anisotropy. ADC quantifies the average dispersion size in each direction, with higher values reflecting less restriction and consequently fewer intact fibers (5). However, ADC is a scalar parameter derived from DWI, assuming isotropic diffusion, typically calculated from signal attenuation across a finite number of diffusion encoding directions. Consequently, ADC exhibits directional dependence, reflecting an apparent diffusion process that integrates intrinsic molecular diffusion, microstructural effects, and acquisition geometry factors. Conversely, MD originates from the diffusion tensor in DTI, and is defined as the arithmetic mean of the tensor's three eigenvalues. MD exhibits rotation invariance, representing the average diffusion coefficient within a voxel, independent of tissue orientation (6). While ADC and MD values may appear similar in isotropic tissues, it is important to note that they are not equivalent in physical essence or computational methodology. In the context of anatomically intricate pelvic structures that may manifest anisotropy, it is imperative to differentiate between ADC and MD. This distinction is crucial to avert conceptual errors and to maintain the interpretability of diffusion indices. In addition to DTI-related parameters, DTI can estimate the overall directionality of muscle fibers based on water diffusivity (7). Using the water molecule data obtained from DTI, several measurements can be computed at both the directional and dispersion levels. The descriptive meanings and clinical interpretations of these measurements are presented in Table 1. It is important to note that each measurement of DTI needs to be interpreted in combination with other imaging and clinical information.

**Table 1:** Description of meaning and clinical interpretation of DTI-related parameters.

DTI indicator	Description of meaning	Clinical interpretation
Fractional anisotropy (FA)	Describing the degree of anisotropy in the diffusion process of water molecules within neural white matter	High FA values: Neatly arranged fibre bundles, etc.
		Low FA values: Muscle damage, inflammation, tumors, etc.
Apparent diffusion coefficient (ADC)	Magnitude of water molecule dispersion, calculated from signal attenuation across a finite number of diffusion encoding directions.	High ADC values: Inflammation, oedema, fatty infiltration, etc.
		Low ADC values: Dense cellularity, fibrosis, etc.
Mean diffusivity (MD)	The average diffusion magnitude regardless of direction, calculated from the arithmetic mean of the three eigenvalues (*λ*1, λ2, λ3) of the diffusion tensor.	High MD values: Inflammation, oedema, necrosis, etc. Low MD values: Dense cellularity, fibrosis, etc.
Axial diffusivity (AD)	Indicates dispersion values parallel to the axon	High AD values: Tissue damage, inflammation, oedema, tumors, etc.
Radial diffusivity (RD)	Indicates dispersion values perpendicular to the axon	Low RD values: Damage in the direction of the fibres

FA, fractional anisotropy; ADC, apparent diffusion coefficient; MD, mean diffusivity; AD, axial diffusivity; RD, radial diffusivity.

DTI has primarily been used to map the brain's microstructure and plays a crucial role in the early detection and treatment of cerebral ischemia (8). Additionally, DTI is frequently applied in the study of peripheral nerves and skeletal muscles (9, 10). However, the application of DTI to abdominal organs remains challenging due to artifacts caused by vascular pulsation, peristalsis, respiration, and the presence of bowel gas (11). In contrast, the negligible respiratory movements in the pelvis, combined with advancements in techniques such as parallel imaging, have made it possible to acquire microstructural information from the pelvic floor (12). To date, studies on pelvic floor DTI have demonstrated that the quantitative parameters provided by this technique offer valuable insights for the clinical diagnosis of disease.

Although DTI has been relatively well established for applications in the brain, peripheral nerves, and skeletal muscle, its systematic implementation in the assessment of pelvic floor structures remains notably limited. Existing studies have largely focused on individual organs or specific pathological conditions, without providing a comprehensive overview of the region. Moreover, to date, no systematic review has comprehensively characterized the imaging features, parameter variations, or clinical applicability of DTI in the pelvic floor. This paper aims to systematically summarize the current state of DTI application in the anal canal, uterus, prostate, and pelvic floor musculature. It further seeks to delineate the imaging characteristics of these structures under both normal and pathological conditions, and to discuss considerations relevant to clinical translation.

## Methods

### Search strategy and information sources

A comprehensive search was conducted in PubMed, EMBASE, and the Cochrane Library using the following search terms, covering the period from the inception of each database through December 11, 2025: (DTI OR diffusion tensor imaging OR diffusion tensor tractography OR diffusion tractography OR fiber tractography) AND (pelvic OR pelvic floor OR pelvic floor dysfunction OR pelvic organ prolapse OR levator ani OR sphincter OR anal canal OR anal fistula OR prostate OR prostate cancer OR uterus OR endometriosis OR fibroid OR uterine fibroid OR bladder OR rectum OR perineum).

### Inclusion criterion

The following inclusion criteria were applied during the systematic evaluation of initial search results:

1. Population: Adults (≥18 years old) with conditions involving pelvic floor structures (rectum, uterus, prostate, pelvic floor muscles) or related diseases.
2. Intervention: The utilisation of DTI as the intervention is hereby proposed.
3. Control: Not mandatory; may include healthy controls, pathological controls, or self-controls.
4. Outcome Measures: Outcome measures include DTI parameters such as FA, ADC, MD, and fiber-tracing results
5. Study Type: RCTs or observational studies (excluding reviews, case reports, conference abstracts, and animal studies).

### Exclusion criteria

The following reasons were used to exclude articles:

1. The phenomenon of duplicate publications
2. The study's design may be considered inappropriate, as may the subject population.
3. The data is either unavailable or irrelevant.

### Selection process

The inclusion and exclusion criteria were applied systematically to all populated articles, which were reviewed in full.

### Data collection process and data items

Data were systematically extracted from the articles and included the following: type of study; treatment prior to recurrence/progression; outcomes pertaining to DTI-related metrics and patterns.

### Synthesis methods and effect measures

All studies included in the analysis provided information on the aforementioned data items and were therefore eligible for synthesis. The results of the data extraction process are summarized in tabular form in each section.

### Risk of bias assessment

All included studies underwent risk of bias assessments. The respective sections provide detailed findings regarding the overall quality of the included articles and their associated risk of bias. The level of evidence for each study was assessed and determined using the Evidence Levels Table developed by the Oxford Centre for Evidence-Based Medicine Evidence Levels Working Group (13). It should be noted that a formal risk-of-bias assessment using dedicated tools was not performed; instead, the quality appraisal relied solely on the OCEBM evidence grading system to categorize the studies based on their design and research questions. Evidence is categorized into five levels (Level 1 being the highest, Level 5 the lowest) in order to evaluate the quality of medical research evidence based on different types of research question. In the course of evaluating each study, the diagnostic question row most relevant to the research question of the study in question was referenced.

## Results

### General findings

**Figure 1:**
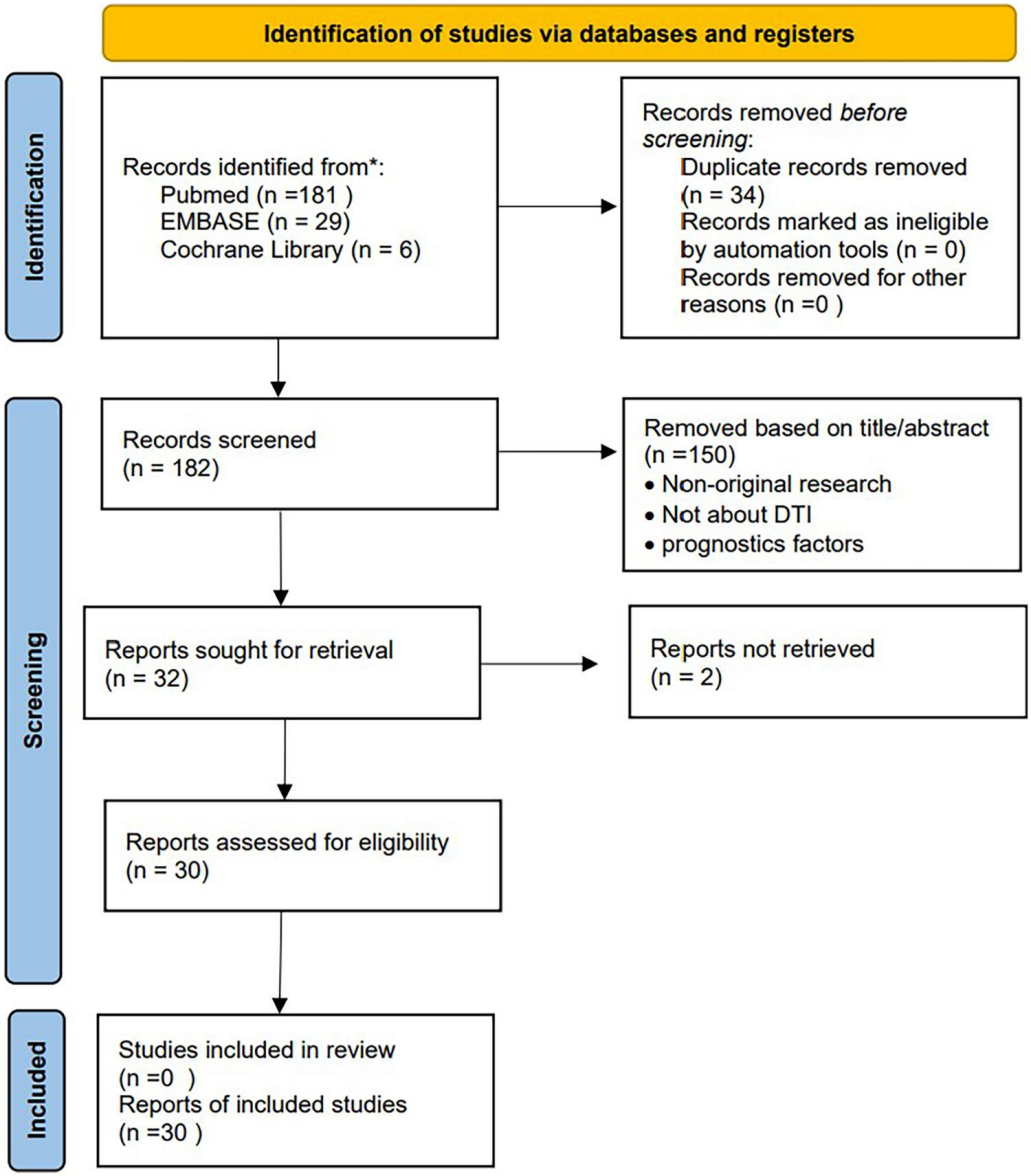
The sequence of steps taken in this systematic review in order to ascertain articles that met the inclusion criteria.

The comprehensive search process yielded a total of 216 results, of which 30 studies satisfied the predetermined inclusion criteria. The process of this systematic review is illustrated in the PRISMA (2020) flow diagram shown in Figure 1 (14). The number of cases included in each study and the level of evidence are detailed in the tables contained in the following sections. Among the 30 included studies, 8 were rated as Level 2, 20 as Level 3, and 1 as Level 4 evidence. Table 2 presents a cross-sectional comparison of different organs, populations, protocols, and clinical findings.

**Table 2:** A cross-sectional comparison of research characteristics across different organs.

Organs/Structure	Number of studies	Populations (Main Categories)	Protocols	Clinical findings
Anal canal	2	Men with prostate cancer; patients with anal fistulas	3T MRI; b values 400–800 s/mm²; ROI delineation by layer (epithelium/IAS/EAS)	FA values vary across layers in a graded manner (highest in the EAS, lowest in the epithelium). Both FA and ADC are reduced in active anal fistulas; DTI shows potential for assessing inflammatory activity.
Uterus	5	Nonpregnant women; females with uterine fibroids or endometriosis; small sample size (primarily <30)	Primarily 3T MRI; b values 700–1,000 s/mm^2^; imaging in three layers (EM/JZ/OM) or of the entire uterus	Uterine fibers run in an inner circular and outer longitudinal pattern. FA values are reduced in the sacral nerve roots of patients with endometriosis. FA values are higher in solid fibroids than in myxous fibroids. DTI can assist with intraoperative navigation.
Prostate	19	Healthy males and males with suspected or confirmed prostate cancer; a wide range of sample sizes (6–364)	Primarily 3T MRI; b values 500–2,500 s/mm^2^;ROI delineation by region (CG/PZ)	FA values in the tumor region vary across different studies (depending on the segmentation method), with MD and ADC generally showing reduced values. DTI can aid in tumor grading, assessment of treatment efficacy, and visualization of nerve tracts.
Pelvic floor muscle	4	Nonpregnant women; females with pelvic organ prolapse; small sample size (5–30)	3T MRI; b values 0–800 s/mm^2^; whole-muscle tracer imaging or targeted ROI delineation	DTI fiber tracking clearly visualizes the puborectalis and levator ani muscles. The FA of the obturator internus muscle was significantly higher in the prolapse group than in the asymptomatic group;

IAS, internal anal sphincter; EAS, external anal sphincter; EM, endometrium; JZ, junctional zone; OM, outer myometrium; CG, central gland; PZ, peripheral zone; ROI, region of interest.

### Anal canal

The anal region is the most distal part of the gastrointestinal (GI) tract, encompassing the anal canal, anal rim, and anal verge (15). The anal canal is composed of three layers: 1) the internal anal sphincter (IAS), a concentric smooth muscle layer of colonic origin that maintains tonic contraction through autonomic innervation; 2) the sphincter gap, which contains the longitudinal muscle coat, vascular plexuses, and neural elements from the pelvic plexus; 3) the external anal sphincter (EAS), a striated voluntary muscle complex divided into subcutaneous, superficial, and deep components, with somatic nerve supply from the pudendal nerve (16, 17).

### Normal stratified structure of the anal canal

**Figure 2:**
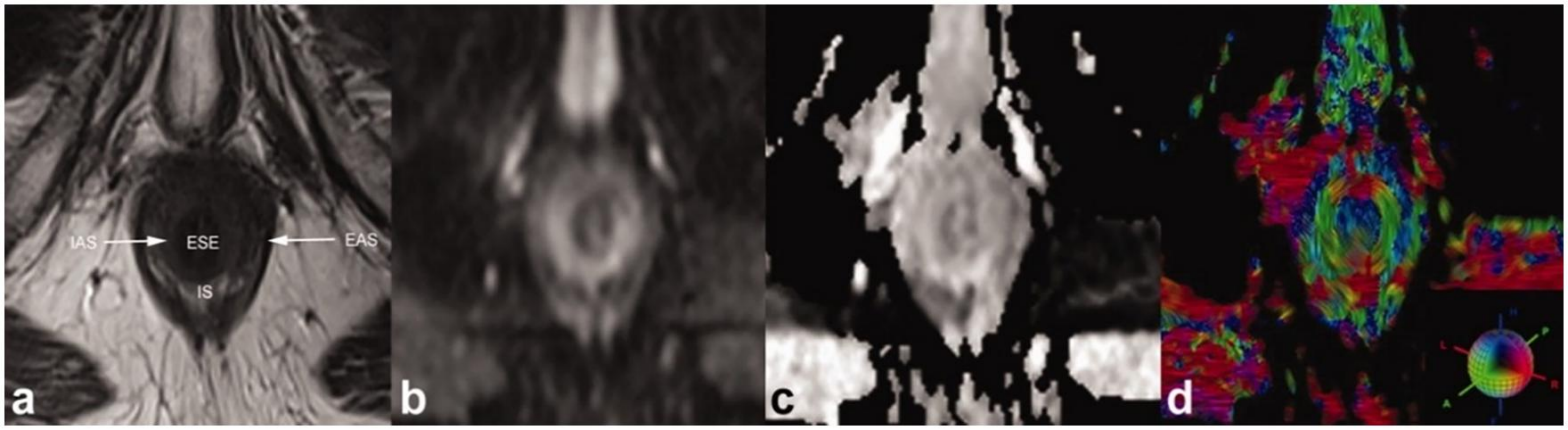
T2-weighted axial image **(a)** and corresponding diffusion weighted b 0 s/mm2 **(b)**, ADC **(c)** and color coded FA **(d)** maps at mid anal canal level. Source: Reprinted from Journal of Magnetic Resonance Imaging (12). Copyright 2025, with permission obtained from Wiley (License No. 6064200283598).

At present, the application of DTI in the anorectal region is still in its early exploratory stages. DTI-based information acquisition in the anal canal focuses on the epithelial/subepithelial layer, the IAS, and the EAS (Figure 2). Goh et al. (12) compared 25 consecutive male patients (mean age of 69 years) with prostate cancer undergoing staging MRI. They found that the FA value of the epithelial/subepithelial layer was the lowest, followed by the IAS, with the EAS showing the highest value. Overall, the FA value of the anal canal was considerably lower than that of reference pelvic musculature. The components of the anal canal exhibit anisotropy, and the measurements of anisotropy can be reliably reproduced. The specific values and results of each measurement are presented in Table 3. The reports and sample sizes on DTI measurements of the anal canal remain relatively small. Future studies should focus on increasing the sample size and defining clear criteria for the collection of DTI-related parameters in the anus of healthy individuals. Global tractography, when used alongside DTI, could provide a better understanding of the function of the anal sphincter. Zifan et al. (17) found that the EAS morphology resembles the number “8” or a “purse string.” Rousset et al. (19) found the mean number of accurate fibers for pubovisceralis was 17 ± 2, 14 ± 6 for the puborectalis, and 1 ± 1 for the iliococcygeus. However, it should be noted that the findings presented here are derived from single-center studies with small sample sizes. Furthermore, there is a lack of uniform threshold values or standardized acquisition and post-processing protocols.

**Table 3:** Human studies investigating DTI in anal canal.

Author (year)	Subjects (number)	Level of evidence	Acquisition parameters	Tissue type	Mean FA (P)	Mean ADC or MD (P) (10 × 3mm2/s)
Goh (12)	men with no anal canal disease（25）	3	TR/TE:2.900/80 ms FOV 260 mm^2^ b values: 0 and 800 mm^2^	Epithelium/subepithelium	0.283 ± 0.099 (0.0001)	MD 1.49 ± 0.23 (0.0001)
				IAS	0.337 ± 0.049 (0.0001)	MD 1.59 ± 0.19 (0.0001)
				EAS	0.415 ± 0.072 (0.0001)	MD 1.51 ± 0.280 (0.0001)
Wang (18)	patients with perianal fistula (34)	3	TR/TE:3.250/48 ms; FOV:200 mm^2^; b values:0 and 400s/mm2	Fistula area in NIA	0.183 ± 0.057 (0.009)	ADC 1.393 ± 0.256 (0.004)
				Fistula area in PIA	0.134 ± 0.046 (0.009)	ADC 0.979 ± 0.441 (0.004)
				Normal area in NIA	0.382 ± 0.084 (0.154)	ADC 1.703 ± 0.432 (0.233)
				Normal area in PIA	0.343 ± 0.070 (0.154)	ADC 1.864 ± 0.336 (0.233)

The ADC or MD values indicated in the table in this article are all based on the actual conditions described in the text.

TR, repetition time; TE, echo time; IAS, internal anal sphincter; EAS, external anal sphincter; NIA, negative inflammation activity; PIA, positive inflammation activity.

### Pathological study: assessment of active anal fistulas

Clinical applications of MRI of the anal canal are commonly used to determine the direction and location of anal fistulas. An anal fistula is a frequent anorectal condition, typically caused by an infected abscess near the rectum and anal canal. Its primary characteristic is the formation of a chronically infected channel connecting the anus or rectum to the perineal skin. Identifying the location and alignment of the fistula tract is crucial, as two important deep posterior interstices in the anal canal are often overlooked or not adequately treated, leading to recurrent fistulas (20). Thus far, the most commonly used MRI technique for anal fistulas is the fast spin echo (TSE) sequence with T1WI, T2WI, FST2WI, and fat-suppressed T1WI for enhancement scans (21, 22), Additionally, it has been reported that DWI is more sensitive in detecting anal fistulas (23, 24). Surprisingly, only one published study has explored the use of DTI in anal fistulas. Wang et al. (18) examined 34 patients with perianal fistulas who underwent routine MRI sequences as well as DTI sequences on a 3.0T MR scanner. They reported the FA values and ADC values for active and inactive anal fistulas. They found that when an anal fistula disrupts the perianal musculature, the FA values decreased. Additionally, the FA and ADC values in the edema area were lower when inflammation was active. Pus, which contains sticky proteins, dead cell debris, bacteria, and immune cells combating the infection, physically obstructs the free movement of water. The high viscosity and abundance of inflammatory cells hinder the diffusion of water molecules, leading to a reduced ADC value. DTI parameters, such as FA and ADC values, may offer insights into the microstructure of perianal fistulas. Given that DTI is more sensitive than DWI, fiber bundle imaging could become a valuable tool for diagnosing anal fistulas in the future. Although preliminary findings from DTI studies in anal fistulas show some promise, its actual diagnostic efficacy has not yet been established. The question of whether DTI can provide independent diagnostic value in comparison to conventional MRI sequences still requires validation through multi-center prospective studies.

### Uterus

The uterus is a fibromuscular organ consisting of three layers: the endometrial, junctional, and myometrial zones. Previous DTI studies have reported that the uterus is made up of an internal circular layer and an external longitudinal layer (25–27). Table 4 provides an overview of human studies evaluating DTI of the uterus.

**Table 4:** Human studies investigating DTI in uterine muscle fiber bundles.

Author (year)	Subjects (number)	Level of evidence	Acquisition parameters	Results
Weiss (25)	Nonpregnant women (5)	3	TR/TE 1,000/60 msec	The uterine cavity: Anisotropic, cyclic
			Matrix 256 × 256	The cervix: outer part is circular, inner part is longitudinal
Fiocchi (26)	Women (30)	2	Long axis: TR/TE 5,000/80 ms	Inner part: circular; Outer part:circular
			Short axis: TR/TE 4,000/80 ms	
			Matrix 256 × 256	

When conducting DTI studies related to the uterus, Clinical attention should be given to the impact of menstrual cycle fluctuations on relevant parameters. A DWI study of the uterus revealed that ADC values fluctuate in accordance with the menstrual cycle and undergo changes within the uterus during the menopausal transition.

### Normal uterine fiber structure and layering characteristics

**Figure 3:**
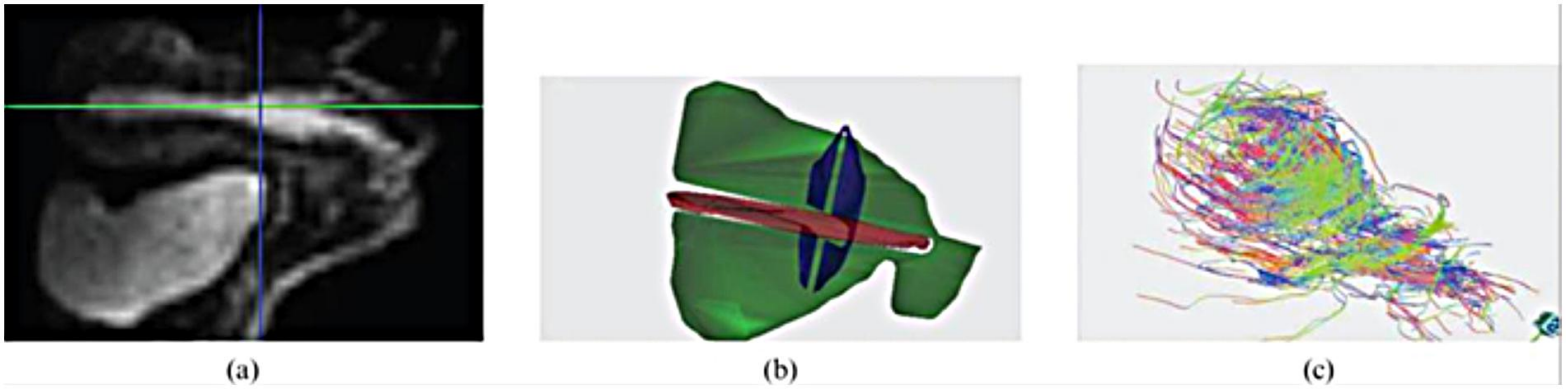
Example of whole-uterus diffusion DTI fibre tracking representation. Regions of interest [sagittal view **(a)**] are drawn to depict fibres of the whole uterus **(b) (c)** Fibre tracking after processing results in global uterus fibre architecture. Source: Reprinted from British Journal of Radiology (26). Copyright 2025, with Permission obtained from Oxford University Press (License No. 6064750085947).

From an anatomical perspective, DTI can provide *in vivo* visualization of the uterus's fiber-related information as well as differences in tissue density across various layers of the uterus. Fiocchi et al. (26) conducted *in vivo* 3T MR-DTI on 30 volunteers and found that the fiber structure of the normal uterus can be mapped *in vivo* using the DTI technique (Figure 3). They observed that in the scarred anterior isthmus, the fiber number and the density of cesarean-scarred uteri were lower than those of nulliparous uteri. However, there was no significant difference in the FA and MD values between the two groups (FA for nulliparous and cesarean-scarred uteri: 0.41 ± 0.02 and 0.42 ± 0.02, *p* = 0.25; MD of nulliparous uteri and cesarean-scarred uteri: 1.93 ± 0.25 × 10–3 mm^2^s^−1^ and 1.82 ± 0.18 × 10–3 mm^2^s^−1^, *p* = 0.20). Fujimoto et al. (28) conducted a study with 10 women and divided the uterus into three layers for measurement: the outer myometrium (OM), the junctional zone (JZ), and the endometrium (EM). They found that the mean FA and MD values in the OM were 0.257 ± 0.022 and 1.12 ± 0.13 × 10–3 mm^2^s^−1^, respectively. In the JZ, the mean FA and MD were 0.297 ± 0.033 and 0.83 ± 0.09 × 10–3 mm^2^s^−1^, and in the EM, the mean FA and MD were 0.186 ± 0.039 and 0.97 ± 0.10 × 10–3 mm^2^s^−1^. The differences in results across reports may be attributed to unstratified analysis and variations in acquisition parameters.

### Pathological study

#### Endometriosis and pelvic pain

With regard to its diagnostic utility, DTI has been shown to differentiate between conditions such as endometriosis and uterine fibroids, as well as to elucidate their pathological mechanisms. Endometriosis is a chronic inflammatory condition where endometrial tissue grows outside the uterus, often accompanied by painful symptoms (29). The central nervous system (CNS) is one of the reasons for pain (30). Manganaro et al. (31, 32) found that endometriosis-related pain is linked to sacral root abnormalities, and DTI can offer a clearer understanding of this pain. DTI revealed that sacral nerve roots (S1, S2, S3) in patients with endometriosis exhibited structural irregularities, disorganized fibre orientation, increased branching, and loss of unidirectionality in three-dimensional fibre tract imaging. Moreover, the FA values in the patient group were found to be significantly lower than those in the healthy control group. Accordingly, DTI can be a powerful tool in the management and therapy for patients with endometriosis.

#### Uterine fibroids and endometrial cancer

Furthermore, a number of studies have investigated the potential of DTI in the diagnosis of uterine fibroids. Uterine fibroids are benign tumors of the uterus and a common indication for hysterectomy (33). Thrippleton et al. (27) conducted *ex vivo* tissue analysis on fibroid uteri and found that FA values were lower in dense fibroid tissue than in the myometrium, but higher than those in myxoid tissue. Toba et al. (34) explored the feasibility of DTI for evaluating myometrial invasion in uterine endometrial cancer. They found that in the FA maps of histopathological stage S and D cancers, a zone of high FA values was present in the myometrium adjacent to the tumor, while no anisotropic zone was observed in stage E cancers.

#### Applications of intraoperative navigation

DTI can also serve as an adjunct in treatment planning. Preliminary studies suggest that DTI fiber tracing can aid in intraoperative decision-making. Chauvet et al. (35) reported two cases in which DTI and fiber tractography were applied during laparoscopic myomectomies to visualize uterine muscle fibers. They demonstrated the potential of using DTI and fiber tractography to determine fiber direction, thereby facilitating the selection of an optimal starting incision point for laparoscopic myomectomy. The specific examination parameters and results from each study are summarized in Table 5.

**Table 5:** Human studies investigating DTI in uterus.

Author (year)	Subjects (number)	Level of evidence	acquisition parameters	Tissue type	Mean FA	Mean ADC or MD (10–3mm2/s)
Fiocchi (26)	Healthy females (30)	2	long axis: TR/TE 5,000/80 ms	Anterior isthmus segment (nonpregnant)	0.41 ± 0.02	MD 1.93 ± 0.25
			short axis: TR/TE 4,000/80 ms	Anterior isthmus segment (caesarean surgery)	0.42 ± 0.02	MD 1.82 ± 0.18
Thrippleton (27)	Females with fibroid tumor of the uterus (9)	3	TR/TE 4,500–5,410/56 msec	Myometrium	0.395 ± 0.032	\
				Fibroid (dense)	0.280 ± 0.053	\
			b values:0 and 915 s/mm^2^	Fibroid (myxoid)	0.201 ± 0.056	\
				Calcification	0.235	\
Fujimoto (28)	Healthy females (9)	3	TR/TE 7.000/62 ms	Outer myometrium	0.257 ± 0.022	MD 1.12 ± 0.13
			b values 0 and 700 s/mm^2^	Junctional zone	0.297 ± 0.033	MD 0.83 ± 0.09
				Inner myometrium	0.186 ± 0.039	MD 0.97 ± 0.10
Toba (34)	Specimens of endometrial cancer (12)	3	b values 0 and 1,000 s/mm^2^	Tumor	0.21 ± 0.05	no difference
				Inner myometrium	0.44 ± 0.01	
				Outer myometrium	0.32 ± 0.08	

#### Prostate

The prostate gland is a substantial, unpaired organ. A normal prostate consists of three main regions: the peripheral zone, the central zone, and the transition zone (36). As men age, the periurethral glandular tissue and transition zone may gradually hypertrophy, compressing the central zone and stretching the peripheral zone. This hyperplasia typically does not affect the peripheral zone; thus, radiologically, only two areas need to be considered: the central gland and the peripheral zone (37).

#### Normal prostate and neurovascular bundle

**Figure 4:**
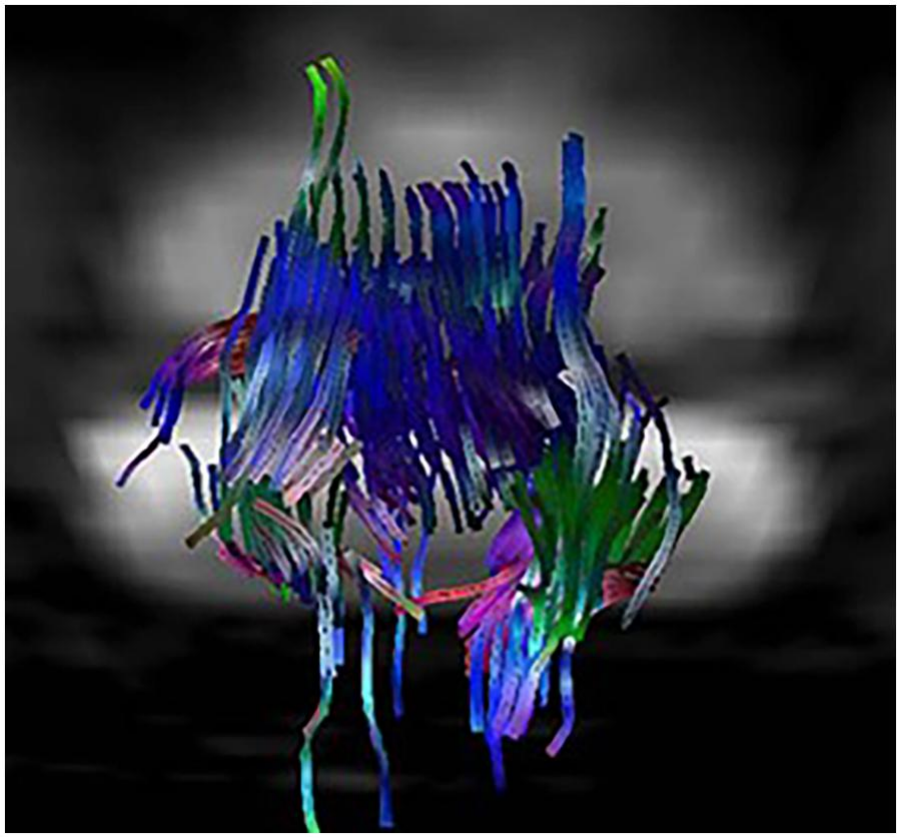
Tractography image of the prostate with dark blue dominancy in the center, and light blue in the periphery, darker color indicated increased anisotropy in the central zone. Source: Reprinted from European Radiology (38). Copyright 2025, with Permission obtained from Springer Nature (License No. 6065940547857).

Most studies have found that the FA value is higher and the ADC value is lower in the central zone than in the peripheral gland in a normal prostate (Figure 4) (38–42). However, Sinha et al. (43) reported that the FA value of the peripheral gland (0.46± 0.04) was higher than that of the central gland (0.40 ± 0.08). The discrepancies in these findings may be due to differences in the parameters used across studies, such as the b values, imaging protocols, technical limitations, and signal-to-noise ratios. Kim et al. (44) found that the number of diffusion-encoding directions did not have a considerable effect on imaging quality in the prostate.

The prostate has a rich neurovascular network. Panebianco et al. (45) demonstrated that DTI can depict the periprostatic plexus in all directions. Finley et al. (46) visualized fiber tracts around the prostate and found that it is feasible to map the periprostatic fiber tract anatomy using DTI tractography. They observed no significant correlation between the total number of tracts and prostate size. The prostate gland consists of a rich neurovascular network, and several studies (45–49) have confirmed that the entire plexus can be described in all directions in the prostate gland with DTI.

### Pathological study

#### Prostate cancer

Prostate cancer is a highly heterogeneous disease, the second most common malignancy worldwide, and the fifth leading cause of cancer deaths in men (50). DTI can be a valuable tool for diagnosing prostate tumors in clinical practice. Significant correlations have been found between DTI and Gleason scores (GS) in assessing tumor aggressiveness in the peripheral zone of prostate cancer (51). Researches have found that when combining the GS scores with DTI metrics to distinguish high-risk from low-risk tumors, both MD values and FA values exhibit systematic differences. Specifically, smaller MD values and larger FA values were both associated with higher cancer risk (52, 53). In other studies (51, 54, 55), researchers found that MD negatively correlated with GS and demonstrated the highest accuracy in distinguishing low-risk from intermediate-to-high-risk prostate cancer within the high b-value range (0–2,500 s/mm²). The analysis of the FA values revealed no statistically significant correlation. The researchers concluded that MD measurement based on high b-value DTI serves as a non-invasive imaging biomarker, aiding in the grading, diagnosis and clinical decision-making for prostate cancer.

Compared to normal tissue, prostate cancer lesions exhibit reduced ADC values and MD values, along with elevated FA values, which can enhance the accuracy of diagnosing prostate tumors at the microstructural level (32, 40, 41, 56–66). Karakoishin et al. (57) found that FA values were significantly higher in central gland cancers and slightly elevated in peripheral zone cancers when compared to normal tissue, based on an analysis of 364 patients with suspected prostate cancer (*P* < 0.0001). However, some studies have reported no statistically significant difference in FA values between tumor and normal regions, likely due to the impact of noise from signal attenuation on FA, which may vary across different areas of the gland (62). By measuring the prostate both *in vivo* and *ex vivo* in post-excision specimens from the same patients, it was found that prostate cancer in the peripheral zone contained a higher cell density, resulting in reduced luminal space and consequently lower MD values (65). At the histopathological level, Hectors et al. (67) found that the MD values from DTI were only related to the stromal fraction. Gholizadeh et al. (58) also found that fiber tract density was higher in cancerous tissue than in healthy tissue, likely due to the increased nerve and vascular density observed in prostate cancer.

#### Assessment of treatment response

DTI can also track changes in prostate structure during therapy. Takayama et al. (64) studied 9 patients with biopsy-proven prostate cancer before and after carbon-ion radiotherapy and found that MD values significantly increased following the therapy. Besides, DTI could detect a decrease in the number of periprostatic neurovascular fibers and FA values after prostatectomy (68, 69). The specific examination parameters and results from each study are summarized in Table 6.

**Table 6:** Human studies investigating DTI in prostate.

Author (year)	Subjects (number)	Level of evidence	Acquisition parameters	Tissue type	Mean FA	Mean ADC or MD (10–3mm^2^/s)
Li (40)	Males with prostate cancer (33)	2	TR/TE 10,000/55 ms	Normal central gland	0.37 ± 0.05	ADC 1.10 ± 0.09
			b values 0 and 700 s/mm^2^	Normal peripheral zone	0.21 ± 0.07	ADC 1.63 ± 0.15
				Cancerous sextants	0.38 ± 0.09	ADC 1.02 ± 0.16
				Non-cancerous sextants	0.31 ± 0.06	ADC 1.22 ± 0.14
Gürses^d^ (38)	Healthy males (30)	3	TR/TE 10,000/53 ms	Central zone	0.260 ± 0.060	ADC 1.220 ± 0.271
			b values 0 and 700 s/mm^2^.	Peripheral zone	0.160 ± 0.030	ADC 1.610 ± 0.347
Gibbs (41)	Males with prostate cancer (26)	3	TR/TE 6,200/64.8 ms	Tumor	0.24 ± 0.05	ADC 1.33 ± 0.32
			b values 0 and 700 s/mm^2^	Normal peripheral Zone	0.16 ± 0.06	ADC 1.86 ± 0.47
Li^e^ (42)	Males with suspected prostate cancer (33)	2	TR/TE 10,000/55 ms	Tumor	0.38 ± 0.09	ADC 1.02 ± 0.16
			b values 0 and 700 s/mm^2^	Normal peripheral Zone	0.3 ± 0.06	ADC 1.22 ± 0.14
Sinha (43)	Healthy males (6)	4	TR/TE 4,000/72 ms	Peripheral Zone (gated)	0.46 ± 0.04	MD 1.95 ± 0.08
			b values 0 and 349.76 s/mm^2^	Peripheral Zone (non-gated)	0.47 ± 0.07	\
				Central Zone (gated)	0.40 ± 0.08	MD 1.53 ± 0.34
				Central Zone (non-gated)	0.48 ± 0.14	\
Kim (44)	Healthy males (12)	3	TR/TE 4,374/68 ms b values 0 and 1,000 s/mm^2^	Central zone (6 directions)	0.34 ± 0.07	MD 1.27 ± 0.09
				Central zone (15 directions)	0.26 ± 0.05	MD 1.25 ± 0.08
				Central zone (32 directions)	0.28 ± 0.05	MD 1.27 ± 0.10
				Peripheral Zone (6 directions)	0.21 ± 0.04	MD 1.46 ± 0.23
				Peripheral Zone (15 directions)	0.15 ± 0.03	MD 1.49 ± 0.23
				Peripheral Zone (32 directions)	0.14 ± 0.02	MD 1.48 ± 0.22
Onay (51)	Males with prostate cancer (38)	2	TR/TE 3,200/(77–81.3)ms	Tumor	0.19 ± 0.05	MD 1.05 ± 0.27
			b values 0 and 800 s/mm^2^	Normal	0.09 ± 0.03	MD 2.02 ± 0.25
Nezzo (54)	Males with prostate cancer (38)	2	TR/TE 3,000/67 ms	Tumor	(b-values 0- 800) 0.30 ± 0.11	MD 0.74 ± 0.15
			b-values 0, 500, 800, 1,000, 1,500, 2,000, 2,500 s/mm^2^	Benign	(b-values 0- 1,500) 0.27 ± 0.10	MD 0.60 ± 0.09
					(b-values 0- 2,500) 0.26 ± 0.08	MD 0.46 ± 0.07
					(b-values 0- 800) 0.22 ± 0.09	MD 1.60 ± 0.27
					(b-values 0- 1,500) 0.22 ± 0.07	MD 1.20 ± 0.19
					(b-values 0- 1,500) 0.21 ± 0.06	MD 0.80 ± 0.11
Manenti (32)	Males with prostate cancer (30)	3	TR/TE 2,500/49 ms	Central Zone	0.41 ± 0.08^c^	MD 1.59 ± 0.40
			b values 0 and 1,000 s/mm^2^	Peripheral Zone	0.47 ± 0.04^c^	MD 1.95 ± 0.38
				Tumor	0.27 ± 0.05	MD 1.06 ± 0.37
Karakoishin (57)	Males with suspected prostate cancer (364)	3	TR/TE 5.396/91.2 ms	Normal central gland	0.214 ± 0.0704	ADC 0.767 ± 0.233
			b values 0 and 600 s/mm^2^	Normal peripheral zone	0.143 ± 0.091	ADC 0.992 ± 0.367
				Cancerous central gland	0.4208 ± 0.1467	ADC 0.549 ± 0.162
				Cancerous peripheral zone	0.381 ± 0.145	ADC 0.535 ± 0.125
Gholizadeh (58)	Males with prostate cancer (11)	3	TR/TE 11,300/101 ms	Normal	0.22 ± 0.04	MD 1.51 ± 0.23
			b values 0 and 1,600 s/mm^2^	Tumor	0.31 ± 0.09	MD 0.99 ± 0.27
Shenhar (59)	Males with suspected prostate cancer (80)	3	TR/TE 4,163/70 ms	Normal central gland	0.34 ± 0.04	MD 1.52 ± 0.12
			b values 0 and 600 s/mm^2^	Normal peripheral zone	0.27 ± 0.06	MD 1.91 ± 0.27
				Cancerous central gland	0.45 ± 0.08	MD 0.85 ± 0.04
				Cancerous peripheral zone	0.38 ± 0.06	MD 1.22 ± 0.28
Kozlowski (60)	Males with prostate cancer (23)	2	TR/TE 2,100/74 ms	Normal central gland	0.18 (0.17–0.22)^a^	MD 1.58 ± 0.11
			b values 0 and 600 s/mm^2^	Normal peripheral zone	0.19 (0.14–0.24)	MD 1.90 ± 0.26
				Cancerous central gland	0.22 ± 0.05	MD 1.38 ± 0.12
				Cancerous peripheral zone	0.20 (0.17–0.25)	MD 1.49 ± 0.24
Kuk^d^ (61)	Males with suspected prostate cancer (31)	2	TR/TE 4,000/6 ms	Tumor	0.68 ± 0.07	MD 1.31 ± 0.34
			b values 0 and 500 s/mm^2^	Normal	0.47 ± 0.07	MD 1.83 ± 0.26
Hedgire (62)	Males with prostate cancer (24)	2	TR/TE 5,000/90.5 ms	Tumor	0.2047^b^	MD 0.0011
			b values 0 and 600 s/mm^2^	Normal	0.2259^b^	MD 0.0014
Uribe^d^ (63)	Males with prostate cancer (13)	3	TR/TE 2,100/36.4 ms	Normal peripheral zone	0.16 ± 0.07	MD 2.07 ± 0.25
			b values 0 and 600 s/mm^2^	Cancerous peripheral zone	0.20 ± 0.07	MD 1.59 ± 0.21
Takayama (64)	Males with prostate cancer (9)	3	TR/TE 2,761/96 ms	Tumor (before therapy)	0.208 ± 0.0407	MD 1.07 ± 0.066
			b values 0 and 700 s/mm^2^	Tumor (after therapy)	0.221 ± 0.0323	MD 1.41 ± 0.0419
Xu (6)	Males with prostate cancer (24)	3	TR/TE 4,000/76 ms	Normal peripheral zone	0.09 ± 0.03	MD 1.66 ± 0.21
			b values 0 and 500 s/mm2	Cancerous peripheral zone	0.14 ± 0.04	MD 0.94 ± 0.14
Gurses^d^ (66)	Males with suspected prostate cancer (25)	2	TR/TE 4,750/80 ms	Tumor	0.301 ± 0.082	MD 1.389 ± 0.221
			b values 0 and 700 s/mm^2^	Prostatitis	0.142 ± 0.04	MD 1.623 ± 0.125
				Normal	0.147 ± 0.048	MD 1.566 ± 0.118

a median (first to third quartile) for non-normally distributed data.

b no significantly difference (*P* = 0.3819).

c no significantly difference (*P* > 0.05).

d The original text does not explicitly distinguish between “ADC” and “MD,” but since it employs a DTI acquisition scheme and simultaneously calculates FA, the ADC values in this literature actually correspond to MD within the DTI framework.

e The original text does not specify how the ADC value is calculated; in this instance, it is assumed that the calculation is performed in the same manner as described in the original text.

Differences in DTI-related values across studies may be attributed to variations in acquisition parameters and the ages of the samples. Caporale et al. (70) found that contrast and discrimination of DTI for prostate cancer were optimized with b values ranging from 0 to 2,000 s/^mm2^, with the best at b = 2,000 s/mm^2^. Bourne et al. (71) observed that MD and FA values exhibited diffusion time dependence in their analysis of three *ex vivo* prostate specimens.

#### Pelvic floor muscle

The pelvic floor muscles consist of a group of muscles and connective tissues situated in the pelvic floor. Their primary role is to support the pelvic organs, such as the bladder, uterus, and rectum, while also aiding in the regulation of urination, defecation, and sexual function. Pelvic floor dysfunction, such as hyperactivity or spasticity of the pelvic floor muscles, as well as weakness or laxity of these muscles, may contribute to chronic pelvic pain. As of now, three DTI studies on the pelvic floor muscles of healthy women have been published.

#### Tracing of normal pelvic floor muscle fibers

**Figure 5:**
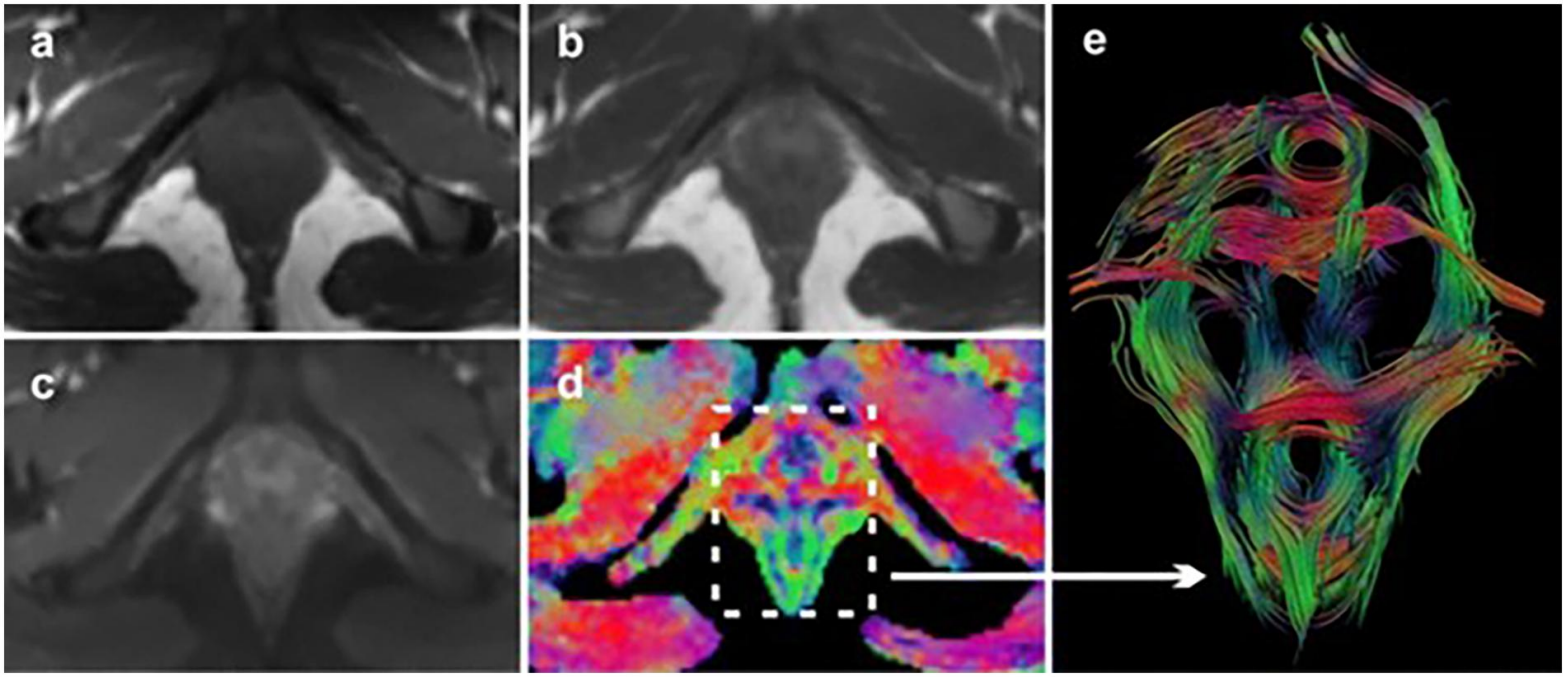
Example of pelvic floor muscle DTI fibre tracking representation. Axial T1-weighted image **(a)**, T2-weighted image **(b)**, b = 0 image **(c)** and corresponding FA map **(d)** With the application of whole volume seeding, present three dimensional (3D) fibre trajectories provide a comprehensive overview of the complex pelvic floor anatomy (caudal view) **(e)** Source: Reprinted from European Radiology (72). Copyright 2025, with Permission obtained from Springer Nature (License No. 6065941156804).

Zijta et al. (72) studied five young nulliparous women and found that fiber tractography effectively provided detailed anatomical visualization of the anal sphincter, urethral sphincter, pubovisceral muscle, perineal body, and internal obturator muscle, with mean FA values of 0.30 ± 0.04, 0.23 ± 0.02, 0.28 ± 0.04, 0.27 ± 0.04, and 0.27 ± 0.01, respectively (Figure 5). Rousset et al. (19) investigated 10 young nulliparous women to explore the architecture of the levator ani complex. They found that fiber tractography performed well for visualizing the pubovisceralis and puborectalis muscles but was inaccurate for the iliococcygeus muscle. Zifan et al. (17) studied 10 healthy nulliparous women and 4 healthy males, revealing that structures such as the bulbospongiosus, ischiocavernosus, transverse perineal muscle, external anal sphincter, and perineal body were clearly visible, with fiber orientation accurately determined through global fiber tracking.

### Pathological study: pelvic floor dysfunction

Pelvic floor dysfunction encompasses a broad range of symptoms, such as incontinence, constipation, difficulty with rectal evacuation, and pelvic organ prolapse. The support of pelvic muscles is essential for maintaining the proper function of pelvic organs (73). Understanding the orientation of skeletal muscle fiber bundles may be valuable in assessing pelvic floor prolapse. Zijta et al. (74) examined 30 women (including 10 with pelvic organ prolapse, 10 with pelvic floor symptoms, and 10 asymptomatic women) using fiber tractography and morphological analysis and found there was a significance difference in FA values for internal obturator muscle between the prolapse group and the asymptomatic group (0.27 ± 0.05 and 0.22 ± 0.03,*P* = 0.015). However, no significant differences were observed in DTI parameters for other muscles, such as the anal sphincter and the pubococcygeus muscle (*P* > 0.1). Although DTI reliably visualizes pelvic muscle structures and exhibits high interobserver agreement, the complexity of muscle architecture may compromise its sensitivity in detecting structural variations. Further research is needed to advance the application of this technique in the field of pelvic floor dysfunction.

## Conclusion

Thus far, the use of DTI has been limited to the anal canal, uterus, prostate, and pelvic floor muscles, and there are fewer studies of DTI in pelvic organs. In most DTI studies on the pelvic floor, the findings align with other structural and functional MRI results, with the orientation of skeletal muscle fiber bundles consistent with current anatomical knowledge. In the same structure, there is a bias in DTI measurements between studies, mainly related to factors such as the scanner, the MRI sequence and its parameters. In order to circumvent image distortion caused by the utilization of excessively high b-values in pursuit of complex tissue microstructures, it is recommended that studies adopt protocols tailored to specific conditions and utilize specific MRI sequences on designated scanners. This methodological approach is instrumental in ensuring a more precise interpretation of the results obtained (75). Furthermore, it is important to acknowledge that low signal-to-noise ratio (SNR) can introduce noise bias, potentially affecting the reliability of both FA and MD measurements during DTI processing. In addition, many studies have limitations due to small sample sizes, varying imaging protocols, or lack of external validation.

Current research indicates that the advantages of applying DTI in the pelvic region include the following. Firstly, it provides quantitative parameters for assessing microstructural disruption in tissues. Secondly, DTI measurements for specific diseases and MR sequences demonstrate reproducibility across specific scanners. Furthermore, DTI has the capacity to enhance diagnostic specificity by distinguishing between benign and malignant or inflammatory lesions through DTI-related parameters. In summary, DTI has the potential to contribute to clinical differentiation and diagnosis of pelvic disorders.

Several limitations of this systematic review should be acknowledged. First, substantial heterogeneity was observed across studies in terms of MRI scanner field strengths (1.5T, 3T, and 7T), b-value selection (ranging from 400 to 2,500 s/mm^2^), methods for ROI delineation, and parameter definitions (e.g., ADC vs. MD), which precluded direct comparisons among studies. Second, the majority of included studies enrolled fewer than 50 participants, and some were case reports or exploratory studies with small sample sizes, thereby limiting the generalizability of the findings. Third, with the exception of prostate cancer, independent external validation cohorts were lacking for most malignancies involving the pelvic floor structures. Additionally, excessive heterogeneity in study designs, outcome measures, and anatomical sites precluded the combination of effect sizes for quantitative synthesis. Finally, the potential for publication bias should be considered, as studies reporting negative or null results may be underrepresented in the literature, which may affect the comprehensiveness of the conclusions. In order to integrate DTI into clinical diagnostic systems, future research should focus on the following: the establishment of multicenter reference value databases; the development of standardized scanning and post-processing protocols; and the exploration of direct correlations between DTI parameters and clinical histopathological changes. Advances in imaging technology and the broader adoption of DTI have led to the anticipation of significant breakthroughs in these areas.

## Data Availability

The original contributions presented in the study are included in the article/Supplementary Material, further inquiries can be directed to the corresponding authors.

## References

[1] Börnert P Norris DG. A half-century of innovation in technology-preparing MRI for the 21st century. Br J Radiol. (2020) 93:20200113. 10.1259/bjr.20200113 32496816 PMC7336051

[2] Basser PJ Pierpaoli C. Microstructural and physiological features of tissues elucidated by quantitative-diffusion-tensor MRI. J Magn Reson. (2011) 213:560–70. 10.1016/j.jmr.2011.09.022 22152371

[3] Beaulieu C. The basis of anisotropic water diffusion in the nervous system - a technical review. NMR Biomed. (2002) 15:435–55. 10.1002/nbm.782 12489094

[4] Arrigoni F Calloni S Huisman T. Conventional MRI. Handb Clin Neurol. (2018) 154:219–34. 10.1016/B978-0-444-63956-1.00013-8 29903441

[5] Schenk P Siebert T Hiepe P Güllmar D Reichenbach JR Wick C. Determination of three-dimensional muscle architectures: validation of the DTI-based fiber tractography method by manual digitization. J Anat. (2013) 223:61–8. 10.1111/joa.12062 23678961 PMC4487763

[6] Basser PJ Mattiello J LeBihan D. Estimation of the effective self-diffusion tensor from the NMR spin echo. J Magn Reson B. (1994) 103:247–54. 10.1006/jmrb.1994.1037 8019776

[7] Brandão S Parente M Silva E Da Roza T Mascarenhas T Leitão J. Pubovisceralis muscle fiber architecture determination: comparison between biomechanical modeling and diffusion tensor imaging. Ann Biomed Eng. (2017) 45:1255–65. 10.1007/s10439-016-1788-y 28097524

[8] Schwarz G Kanber B Prados F Browning S Simister R Jäger HR. Whole-brain diffusion tensor imaging predicts 6-month functional outcome in acute intracerebral haemorrhage. J Neurol. (2023) 270:2640–8. 10.1007/s00415-023-11592-7 36806785 PMC10129992

[9] Simon NG Lagopoulos J Gallagher T Kliot M Kiernan MC. Peripheral nerve diffusion tensor imaging is reliable and reproducible. J Magn Reson Imaging. (2016) 43:962–9. 10.1002/jmri.25056 26397723

[10] Martín-Noguerol T Barousse R Wessell DE Rossi I Luna A. Clinical applications of skeletal muscle diffusion tensor imaging. Skeletal Radiol. (2023) 52:1639–49. 10.1007/s00256-023-04350-3 37083977

[11] Lanzman RS Wittsack HJ. Diffusion tensor imaging in abdominal organs. NMR Biomed. (2017) 30:e3434. 10.1002/nbm.3434 26556181

[12] Goh V Tam E Taylor NJ Stirling JJ Simcock IC Jones RG. Diffusion tensor imaging of the anal canal at 3 tesla: feasibility and reproducibility of anisotropy measures. J Magn Reson Imaging. (2012) 35:820–6. 10.1002/jmri.22873 22127778

[13] OCEBM Levels of Evidence Working Group. The Oxford 2011 Levels of Evidence. Oxford: Oxford Centre for Evidence-Based Medicine (2011). Available online at: http://www.cebm.net/index.aspx?o=5653

[14] Tugwell P Tovey D. RISMA 2020. J Clin Epidemiol. (2021) 134:A5–6. 10.1016/j.jclinepi.2021.04.008 34016443

[15] Lawson JO. Pelvic anatomy. II. Anal canal and associated sphincters. Ann R Coll Surg Engl. (1974) 54:288–300. 4833780 PMC2388404

[16] Barleben A Mills S. Anorectal anatomy and physiology. Surg Clin North Am. (2010) 90:1–15. 10.1016/j.suc.2009.09.001 20109629

[17] Zifan A Reisert M Sinha S Ledgerwood-Lee M Cory E Sah R. Connectivity of the superficial muscles of the human perineum: a diffusion tensor imaging-based global tractography study. Sci Rep. (2018) 8:17867. 10.1038/s41598-018-36099-4 30552351 PMC6294750

[18] Wang Y Gu C Huo Y Han W Yu J Ding C. Diffusion tensor imaging for evaluating perianal fistula: feasibility study. Medicine (Baltimore). (2018) 97:e11570. 10.1097/MD.0000000000011570 30024560 PMC6086465

[19] Rousset P Delmas V Buy J-N Rahmouni A Vadrot D Deux J-F. *In vivo* visualization of the levator ani muscle subdivisions using MR fiber tractography with diffusion tensor imaging. J Anat. (2012) 221:221–8. 10.1111/j.1469-7580.2012.01538.x 22757638 PMC3458627

[20] Zhang H Zhou Z- Hu B Liu D- Peng H Xie S- Clinical significance of 2 deep posterior perianal spaces to Complex cryptoglandular fistulas. Dis Colon Rectum. (2016) 59:766–74. 10.1097/DCR.0000000000000628 27384095

[21] Spencer JA Ward J Beckingham IJ Adams C Ambrose NS. Dynamic contrast-enhanced MR imaging of perianal fistulas. AJR Am J Roentgenol. (1996) 167:735–41. 10.2214/ajr.167.3.8751692 8751692

[22] Torkzad MR Karlbom U. MRI For assessment of anal fistula. Insights Imaging. (2010) 1:62–71. 10.1007/s13244-010-0022-y 22347906 PMC3259332

[23] Cavusoglu M Duran S Sözmen Cılız D Tufan G Hatipoglu Çetin HG Ozsoy A. Added value of diffusion-weighted magnetic resonance imaging for the diagnosis of perianal fistula. Diagn Interv Imaging. (2017) 98:401–8. 10.1016/j.diii.2016.11.002 27964847

[24] Dohan A Eveno C Oprea R Pautrat K Placé V Pocard M. Diffusion-weighted MR imaging for the diagnosis of abscess complicating fistula-in-ano: preliminary experience. Eur Radiol. (2014) 24:2906–15. 10.1007/s00330-014-3302-y 25038854

[25] Weiss S Jaermann T Schmid P Staempfli P Boesiger P Niederer P. Three-dimensional fiber architecture of the nonpregnant human uterus determined *ex vivo* using magnetic resonance diffusion tensor imaging. Anat Rec A Discov Mol Cell Evol Biol. (2006) 288:84–90. 10.1002/ar.a.20274 16345078

[26] Fiocchi F Nocetti L Siopis E Currà S Costi T Ligabue G. *In vivo* 3T MR diffusion tensor imaging for detection of the fibre architecture of the human uterus: a feasibility and quantitative study. Br J Radiol. (2012) 85:e1009–17. 10.1259/bjr/76693739 22744322 PMC3500798

[27] Thrippleton MJ Bastin ME Munro KI Williams AR Oniscu A Jansen MA. *Ex vivo* water diffusion tensor properties of the fibroid uterus at 7T and their relation to tissue morphology. J Magn Reson Imaging. (2011) 34:1445–51. 10.1002/jmri.22793 21953730

[28] Fujimoto K Kido A Okada T Uchikoshi M Togashi K. Diffusion tensor imaging (DTI) of the normal human uterus *in vivo* at 3 tesla: comparison of DTI parameters in the different uterine layers. J Magn Reson Imaging. (2013) 38:1494–500. 10.1002/jmri.24114 23576451

[29] Chapron C Marcellin L Borghese B Santulli P. Rethinking mechanisms, diagnosis and management of endometriosis. Nat Rev Endocrinol. (2019) 15:666–82. 10.1038/s41574-019-0245-z 31488888

[30] Stratton P Berkley KJ. Chronic pelvic pain and endometriosis: translational evidence of the relationship and implications. Hum Reprod Update. (2011) 17:327–46. 10.1093/humupd/dmq050 21106492 PMC3072022

[31] Manganaro L Porpora MG Vinci V Bernardo S Lodise P Sollazzo P. Diffusion tensor imaging and tractography to evaluate sacral nerve root abnormalities in endometriosis-related pain: a pilot study. Eur Radiol. (2014) 24:95–101. 10.1007/s00330-013-2981-0 23982288

[32] Manenti G Carlani M Mancino S Colangelo V Di Roma M Squillaci E. Diffusion tensor magnetic resonance imaging of prostate cancer. Invest Radiol. (2007) 42:412–9. 10.1097/01.rli.0000264059.46444.bf 17507813

[33] Stewart EA. Uterine fibroids. Lancet. (2001) 357:293–8. 10.1016/S0140-6736(00)03622-9 11214143

[34] Toba M Miyasaka N Sakurai U Yamada I Eishi Y Kubota T. Diagnostic possibility of diffusion tensor imaging for the evaluation of myometrial invasion in endometrial cancer: an *ex vivo* study. J Magn Reson Imaging. (2011) 34:616–22. 10.1002/jmri.22693 21751283

[35] Chauvet P Bourdel N Calvet L Magnin B Teluob G Canis M. Augmented reality with diffusion tensor imaging and tractography during laparoscopic myomectomies. J Minim Invasive Gynecol. (2020) 27:973–6. 10.1016/j.jmig.2019.11.007 31765829

[36] McNeal JE. Normal histology of the prostate. Am J Surg Pathol. (1988) 12:619–33. 10.1097/00000478-198808000-00003 2456702

[37] Coakley FV Hricak H.: radiologic anatomy of the prostate gland: a clinical approach. Radiol Clin North Am. (2000) 38:15–30. 10.1016/S0033-8389(05)70147-0 10664664

[38] Gürses B Kabakci N Kovanlikaya A Firat Z Bayram A Uluð AM. Diffusion tensor imaging of the normal prostate at 3 Tesla. Eur Radiol. (2008) 18:716–21. 10.1007/s00330-007-0795-7 17960389

[39] desouza NM Reinsberg SA Scurr ED Brewster JM Payne GS. Magnetic resonance imaging in prostate cancer: the value of apparent diffusion coefficients for identifying malignant nodules. Br J Radiol. (2007) 80:90–5. 10.1259/bjr/24232319 17303616

[40] Li C Chen M Li S Zhao X Zhang C Liu M. Diffusion tensor imaging of prostate at 3.0 Tesla. Acta Radiol. (2011) 52:813–7. 10.1258/ar.2011.100487 21586608

[41] Gibbs P Pickles MD Turnbull LW. Diffusion imaging of the prostate at 3.0 Tesla. Invest Radiol. (2006) 41:185–8. 10.1097/01.rli.0000192418.30684.14 16428991

[42] Li C Chen M Li S Zhao X Zhang C Luo X. Detection of prostate cancer in peripheral zone: comparison of MR diffusion tensor imaging, quantitative dynamic contrast-enhanced MRI, and the two techniques combined at 3.0T. Acta Radiol. (2014) 55:239–47. 10.1177/0284185113494978 23892233

[43] Sinha S Sinha U. *In vivo* diffusion tensor imaging of the human prostate. Magn Reson Med. (2004) 52:530–7. 10.1002/mrm.20190 15334571

[44] Kim CK Jang SM Park BK. Diffusion tensor imaging of normal prostate at 3T: effect of number of diffusion-encoding directions on quantitation and image quality. Br J Radiol. (2012) 85:E279–83. 10.1259/bjr/21316959 21896666 PMC3474052

[45] Panebianco V Barchetti F Sciarra A Marcantonio A Zini C Salciccia S. *In vivo* 3D neuroanatomical evaluation of periprostatic nerve plexus with 3T-MR diffusion tensor imaging. Eur J Radiol. (2013) 82:1677–82. 10.1016/j.ejrad.2013.05.013 23773553

[46] Finley DS Ellingson BM Natarajan S Zaw TM Raman SS Schulam P. Diffusion tensor magnetic resonance tractography of the prostate: feasibility for mapping periprostatic fibers. Urology. (2012) 80:219–23. 10.1016/j.urology.2012.03.027 22748877

[47] Siracusano S Porcaro AB Tafuri A Pirozzi M Cybulski A Shakir A. Visualization of peri-prostatic neurovascular fibers before and after radical prostatectomy by means of diffusion tensor imaging (DTI) with clinical correlations: preliminary report. J Robot Surg. (2020) 14:357–63. 10.1007/s11701-019-00998-z 31280463

[48] Sievert K-D Hennenlotter J Dillenburg T Toomey P Wöllner J Zweers P. Extended periprostatic nerve distributions on the prostate surface confirmed using diffusion tensor imaging. BJU Int. (2019) 123:995–1004. 10.1111/bju.14508 30091828

[49] Di Paola V Totaro A Gui B Miccò M Rodolfino E Avesani G. Depiction of periprostatic nerve fibers by means of 1.5T diffusion tensor imaging. Abdom Radiol. (2021) 46:2760–9. 10.1007/s00261-020-02682-5 32737544

[50] Bray F Laversanne M Sung H Ferlay J Siegel RL Soerjomataram I. Global cancer statistics 2022: GLOBOCAN estimates of incidence and mortality worldwide for 36 cancers in 185 countries. CA Cancer J Clin. (2024) 74:229–63. 10.3322/caac.21834 38572751

[51] Onay A Ertas G Vural M Acar O Saglican Y Coskun B. Evaluation of peripheral zone prostate cancer aggressiveness using the ratio of diffusion tensor imaging measures. Contrast Media Mol Imaging. (2017) 2017(1):5678350. 10.1155/2017/5678350 29097929 PMC5635474

[52] Tian W Zhang J Tian F Shen J Niu T He G. Correlation of diffusion tensor imaging parameters and Gleason scores of prostate cancer. Exp Ther Med. (2018) 15:351–6. 10.3892/etm.2017.5363 29250155 PMC5729705

[53] Ertas G. Detection of high GS risk group prostate tumors by diffusion tensor imaging and logistic regression modelling. Magn Reson Imaging. (2018) 50:125–33. 10.1016/j.mri.2018.04.003 29649574

[54] Nezzo M Di Trani MG Caporale A Miano R Mauriello A Bove P. Mean diffusivity discriminates between prostate cancer with grade group 1&2 and grade groups equal to or greater than 3. Eur J Radiol. (2016) 85:1794–801. 10.1016/j.ejrad.2016.08.001 27666618

[55] Di Trani MG Nezzo M Caporale AS De Feo R Miano R Mauriello A. Performance of diffusion kurtosis imaging versus diffusion tensor imaging in discriminating between benign tissue, low and high Gleason grade prostate cancer. Acad Radiol. (2019) 26:1328–37. 10.1016/j.acra.2018.11.015 30545680

[56] Park SY Kim CK Park BK Ha SY Kwon GY Kim B. Diffusion-tensor MRI at 3T: differentiation of central gland prostate cancer from benign prostatic hyperplasia. AJR Am J Roentgenol. (2014) 202:W254–262. 10.2214/AJR.13.11015 24555622

[57] Karakoishin K Zholdybay Z Ainakulova A Dauytova Y Kamhen V. Comparative analysis of the apparent diffusion coefficient and diffusion tensor imaging in the diagnosis of prostate cancer. Asian Pacific J Cancer Prev. (2024) 25:2397–408. 10.31557/APJCP.2024.25.7.2397 PMC11480594 39068573

[58] Gholizadeh N Greer PB Simpson J Denham J Lau P Dowling J. Characterization of prostate cancer using diffusion tensor imaging: a new perspective. Eur J Radiol. (2019) 110:112–20. 10.1016/j.ejrad.2018.11.026 30599846

[59] Shenhar C Degani H Ber Y Baniel J Tamir S Benjaminov O. Diffusion is directional: innovative diffusion tensor imaging to improve prostate cancer detection. Diagnostics. (2021) 11:563. 10.3390/diagnostics11030563 33804783 PMC8003841

[60] Kozlowski P Chang SD Jones EC Goldenberg SL. Assessment of the need for DCE MRI in the detection of dominant lesions in the whole gland: correlation between histology and MRI of prostate cancer. NMR Biomed. (2018) 31:e3882. 10.1002/nbm.3882 29266527

[61] Yoon SK Kim DW Ha DH Kwon HJ Kang M Choi S. Value of diffusion tensor imaging of prostate cancer: comparison with systemic prostate biopsy. J Korean Soc Radiol. (2011) 64:179–84. 10.3348/jksr.2011.64.2.179

[62] Hedgire S Tonyushkin A Kilcoyne A Efstathiou JA Hahn PF Harisinghani M. Quantitative study of prostate cancer using three dimensional fiber tractography. World J Radiol. (2016) 8:397–402. 10.4329/wjr.v8.i4.397 27158426 PMC4840197

[63] Uribe CF Jones EC Chang SD Goldenberg SL Reinsberg SA Kozlowski P. *In vivo* 3T and *ex vivo* 7T diffusion tensor imaging of prostate cancer: correlation with histology. Magn Reson Imaging. (2015) 33:577–83. 10.1016/j.mri.2015.02.022 25721995 PMC5462375

[64] Takayama Y Kishimoto R Hanaoka S Nonaka H Kandatsu S Tsuji H. ADC Value and diffusion tensor imaging of prostate cancer: changes in carbon-ion radiotherapy. J Magn Reson Imaging. (2008) 27:1331–5. 10.1002/jmri.21388 18504751

[65] Xu J Humphrey PA Kibel AS Snyder AZ Narra VR Ackerman JJH. Magnetic resonance diffusion characteristics of histologically defined prostate cancer in humans. Magn Reson Med. (2009) 61:842–50. 10.1002/mrm.21896 19215051 PMC3080096

[66] Gürses B Tasdelen N Yencilek F Kılıckesmez NO Alp T Fırat Z. Diagnostic utility of DTI in prostate cancer. Eur J Radiol. (2011) 79:172–6. 10.1016/j.ejrad.2010.01.009 20138721

[67] Hectors SJ Semaan S Song C Lewis S Haines GK Tewari A. Advanced diffusion-weighted imaging modeling for prostate cancer characterization: correlation with quantitative histopathologic tumor tissue composition-A hypothesis-generating study. Radiology. (2018) 286:938–48. 10.1148/radiol.2017170904 29117481

[68] Di Paola V Cybulski A Belluardo S Cavicchioli F Manfredi R Pozzi Mucelli R. Evaluation of periprostatic neurovascular fibers before and after radical prostatectomy by means of 1.5T MRI diffusion tensor imaging. Br J Radiol. (2018) 91:20170318. 10.1259/bjr.20170318 29388808 PMC6190772

[69] Kitajima K Takahashi S Ueno Y Miyake H Fujisawa M Sugimura K. Visualization of periprostatic nerve fibers before and after radical prostatectomy using diffusion tensor magnetic resonance imaging with tractography. Clin Imaging. (2014) 38:302–6. 10.1016/j.clinimag.2014.01.009 24629793

[70] Caporale AS Nezzo M Di Trani MG Maiuro A Miano R Bove P. Acquisition parameters influence diffusion metrics effectiveness in probing prostate tumor and age-related microstructure. J Pers Med. (2023) 13:860. 10.3390/jpm13050860 37241031 PMC10222109

[71] Bourne R Liang S Panagiotaki E Bongers A Sved P. Measurement and modeling of diffusion time dependence of apparent diffusion coefficient and fractional anisotropy in prostate tissue *ex vivo*. NMR Biomed. (2017) 30:e3751. 10.1002/nbm.3751 28665041

[72] Zijta FM Froeling M Van Der Paardt MP Lakeman MME Bipat S Montauban van Swijndregt AD. Feasibility of diffusion tensor imaging (DTI) with fibre tractography of the normal female pelvic floor. Eur Radiol. (2011) 21:1243–9. 10.1007/s00330-010-2044-8 21197534 PMC3088829

[73] DeLancey JO. What’s new in the functional anatomy of pelvic organ prolapse? Curr Opin Obstet Gynecol. (2016) 28:420–9. 10.1097/GCO.0000000000000312 27517338 PMC5347042

[74] Zijta FM Lakeman MME Froeling M Van Der Paardt MP Borstlap CSV Bipat S. Evaluation of the female pelvic floor in pelvic organ prolapse using 3.0-Tesla diffusion tensor imaging and fibre tractography. Eur Radiol. (2012) 22:2806–13. 10.1007/s00330-012-2548-5 22797954 PMC3486990

[75] Jones DK Basser PJ. Squashing peanuts and smashing pumpkins": how noise distorts diffusion-weighted MR data. Magn Reson Med. (2004) 52:979–93. 10.1002/mrm.20283 15508154

